# Upregulation of CYP17A1 by Sp1-mediated DNA demethylation confers temozolomide resistance through DHEA-mediated protection in glioma

**DOI:** 10.1038/oncsis.2017.31

**Published:** 2017-05-22

**Authors:** J-Y Chuang, W-L Lo, C-Y Ko, S-Y Chou, R-M Chen, K-Y Chang, J-J Hung, W-C Su, W-C Chang, T-I Hsu

**Affiliations:** 1 The Ph.D. Program for Neural Regenerative Medicine, College of Medical Science and Technology, Taipei Medical University and National Health Research Institutes, Taipei, Taiwan; 2 Comprehensive Cancer Center, Taipei Medical University, Taipei, Taiwan; 3 Division of Neurosurgery, Taipei Medical University-Shuang-Ho Hospital, Taipei, Taiwan; 4 Graduate Institute of Medical Sciences, College of Medicine, Taipei Medical University, Taipei, Taiwan; 5 National Institute of Cancer Research, National Health Research Institutes, Zhunan, Taiwan; 6 Department of Biotechnology and Bioindustry Sciences, College of Bioscience and Biotechnology, National Cheng Kung University, Tainan, Taiwan; 7 Department of Internal Medicine, National Cheng Kung University Hospital, Tainan, Taiwan; 8 Center for Neurotrauma and Neuroregeneration, College of Medical Science and Technology, Taipei Medical University, Taipei, Taiwan

## Abstract

Steroidogenesis-mediated production of neurosteroids is important for brain homeostasis. Cytochrome P450 17A1 (CYP17A1), which converts pregnenolone to dehydroepiandrosterone (DHEA) in endocrine organs and the brain, is required for prostate cancer progression and acquired chemotherapeutic resistance. However, whether CYP17A1-mediated DHEA synthesis is involved in brain tumor malignancy, especially in glioma, the most prevalent brain tumor, is unknown. To investigate the role of CYP17A1 in glioma, we determined that CYP17A1 expression is significantly increased in gliomas, which secrete more DHEA than normal astrocytes. We found that as gliomas became more malignant, both CYP17A1 and DHEA were significantly upregulated in temozolomide (TMZ)-resistant cells and highly invasive cells. In particular, the increase of CYP17A1 was caused by Sp1-mediated DNA demethylation, whereby Sp1 competed with DNMT3a for binding to the CYP17A1 promoter in TMZ-resistant glioma cells. CYP17A1 was required for the development of glioma cell invasiveness and resistance to TMZ-induced cytotoxicity. In addition, DHEA markedly attenuated TMZ-induced DNA damage and apoptosis. Together, our results suggest that components of the Sp1–CYP17A1–DHEA axis, which promotes the development of TMZ resistance, may serve as potential biomarkers and therapeutic targets in recurrent glioma.

## Introduction

Neurosteroids, steroid hormones synthesized by steroidogenesis in the brain, regulate the functions and development of the central nervous system.^1^ The brain is a cholesterol-rich organ that expresses several steroidogenic enzymes, such as cytochrome P450 11A1 (CYP11A1), 17A1 (CYP17A1) and steroidogenic acute regulatory protein.^1^ The regulation of neurosteroid biosynthesis is important for neural homeostasis. The neuroprotective effect of the neural androgen dehydroepiandrosterone (DHEA) has been demonstrated to attenuate oxidative stress and prevent neurodegenerative disorders.^2, 3^ However, whether DHEA affects the behavior of astrocytes, and even astrocyte-derived glioblastomas (GBMs), has not been adequately studied.

Previously, steroidogenesis has been shown to promote the development of adrenocortical, endometrial and prostate cancers, all of which exhibit abundant steroidogenesis.^4, 5, 6^ Evidence that CYP17A1 is absolutely required for steroidogenesis in the prostate gland suggests that CYP17A1 may be a valuable therapeutic target in prostate cancer.^7^ In contrast, whether neural DHEA and steroidogenesis are involved in the development of brain tumors that utilize high levels of cholesterol,^8^ such as GBM, is unknown. Neural allopregnanolone promotes the proliferation of neural progenitor cells,^9^ thus raising the possibility that neurosteroids may induce aberrant proliferation and even tumorigenesis in the brain. Furthermore, to dissect the mechanism underlying expression of the steroidogenic enzyme CYP17A1 in the brain is a priority to evaluate the role of DHEA in GBM.

GBM is a highly aggressive brain tumor for which no effective treatment is available. The standard care protocol of patients with GBM consists of surgical resection followed by radio and chemotherapy.^10, 11^ Unfortunately, temozolomide (TMZ), the major chemotherapeutic drug for the treatment of GBM, does not benefit all patients, and GBM tumors always develop resistance to TMZ.^11^ The main mechanisms of TMZ resistance have been identified, including the existence of cancer stem-like cells and the expression of methylguanine DNA methyltransferase (MGMT).^11^ However, GBM can develop resistance via an MGMT-independent pathway,^12, 13^ thus suggesting the existence of an unknown mechanism for resistance to TMZ. In prostate cancer, in which survival is highly dependent on steroidogenesis, CYP17A1 promotes prostate cancer progression and reduces the chemotherapeutic efficiency.^6, 14, 15^ In brain tissue, CYP17A1-mediated DHEA production may promote GBM to become more malignant and resistant to TMZ.

Specificity protein (Sp) 1 has been reported to regulate the expression of enzymes involved in steroid hormone biosynthesis, such as aromatase.^16^ Sp1 promotes the expression of CYP11A1, which converts cholesterol to pregnenolone, by cooperating with other factors, such as SF-1 and AP-2 in adrenal cortical and trophoblastic cells, respectively.^17, 18^ However, beyond CYP11A1, other key steroidogenic enzymes regulated by Sp1 have not been identified. Multiple functions of Sp1 in tumor biology have been well studied, including in cell cycle progression, angiogenesis, apoptosis, DNA damage and metastasis.^19^ Nevertheless, whether Sp1’s regulation of cholesterol metabolism promotes glioma progression is unknown. Therefore, we focused on studying the effects of Sp1 on CYP17A1 expression and DHEA production in GBM tumors. In particular, whether Sp1-mediated CYP17A1 expression induces TMZ resistance through the action of DHEA has not been determined.

Herein, we report that Sp1 enhanced GBM invasiveness and increased DHEA production by increasing CYP17A1 expression. In particular, Sp1 inhibited DNMT3a-mediated DNA methylation of the CYP17A1 promoter, thereby leading to CYP17A1 upregulation, which then prevented TMZ-induced apoptosis. Together, our results indicate that the activation of the Sp1–CYP17A1 axis contributes to the malignant process of GBM by increasing DHEA production.

## Results

### CYP17A1-mediated DHEA synthesis promotes glioma development and TMZ resistance

**Figure 1:**
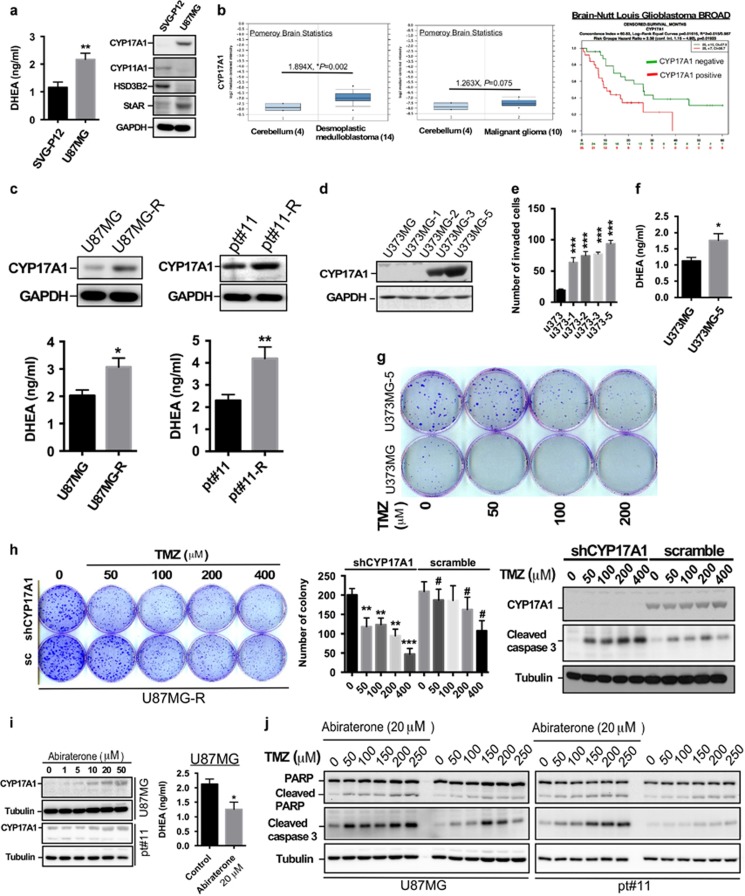
Both CYP17A1 expression and DHEA secretion are increased in malignant GBM. (**a**) The medium from SVG-P12 and U87MG cells were collected and subjected to DHEA ELISA. Data are expressed as the means±s.e.m. (**P<0.01). The cell lysates were subjected to western blotting. (**b**) CYP17A1 mRNA expression was analyzed by the oncomine website and the correlation with patient survival was analyzed by the SurvExpress website. (**c**) Upper panel: western blotting for CYP17A1. Lower panel: the cultured medium was collected and subjected to ELISA. The experiments were performed independently three times, and the data are expressed as the means±s.e.m. (*P<0.05, **P<0.01). (**d**) Western blot of CYP17A1 expression. (**e**, **f**) U373MG cell lines were subjected to invasion and colony formation assays. (**g**) Effect of TMZ on cell survival characterized by colony formation assay. (**h**) After CYP17A1 knockdown, a colony formation assay (left panel) with U87MG-R cells was performed and quantified (middle panel). Data are expressed as the means ± s.e.m. (***P<0.001), #*P*<0.05 is the comparison between samble and shCYP17A1 at the same dose of TMZ. Subsequently, the cells were collected and subjected to western blotting with anti-CYP17A1 or anti-caspase antibodies (right panel). (**i**) Left, effect of abiraterone on CYP17A1 expression; right, after treatment for 24 h, media from U87MG was collected for ELISA. (**j**) After pretreatment with 20 μm abiraterone for 24 h, cells were treated with TMZ for the additional 24 h. Lysates were collected for western blotting. ELISA, enzyme-linked immunosorbent assay.

Neurosteroids are required for the homeostasis of brain functions, and the study of the actions of neurosteroids, such as DHEA, has mainly focused on neuronal protection. There is no evidence indicating that steroidogenesis regulates astrocyte proliferation, even aberrant hyperplasia and glioma development. To investigate whether DHEA is involved in glioma development, we estimated the level of DHEA in human serum. The DHEA levels in glioma patients were significantly higher than those in the normal population (Supplementary Figure S1 and Supplementary Table S1). We next examined which steroidogenic enzyme mediates increased DHEA production in glioma patients. Multiple steroidogenic enzymes, including CYP17A1, are shown to be expressed in different areas of brain, such as amygdala, cerebellum, corpus callosum and hippocampus.^20^ CYP17A1 also aggressively participates in steroidogenesis in brain.^21^ Therefore, before we studied the role of CYP17A1-mediated steroidogenesis in glioma, we also confirmed the expression of CYP17A1 in immortalized human astrocyte, SVG-P12, GBM cell line, U87MG and human brain tissue (Supplementary Figure S2). Compared with HeLa cell served as the positive control, CYP17A1 were expressed in astrocytes, GBM and human brain tissue. Further, U87MG cells obviously expressed the higher level of CYP17A1, compared with SVG-P12 and human brain tissue. This result suggests that CYP17A1 may participate in glioma development. In addition, we found that in U87MG cells, compared with SVG-P12 astrocytes, CYP17A1, but not CYP11A1 or HSD3B2, was significantly increased; moreover, U87MG cells secreted more DHEA (Figure 1a). Therefore, we hypothesized that CYP17A1 might play an important role in GBM development. In the oncomine database, CYP17A1 messenger RNA (mRNA) was significantly increased in brain tumor tissues compared with normal brain tissues (Figure 1b). Importantly, CYP17A1 significantly correlated with a poor prognosis of GBM (Figure 1b). These results suggest that the CYP17A1–DHEA axis participates in glioma development. To determine whether the CYP17A1–DHEA axis is indeed involved in the process of glioma malignancy, we established more aggressive glioma cell lines that exhibited resistance to TMZ or became more invasive. As shown in Supplementary Figure S3 and Figure 1c, both CYP17A1 expression and DHEA secretion were markedly increased in resistant U87MG, and pt#11, which was derived from a GBM patient. Moreover, we selected U373MG-series cell lines possessing different invasive activity by using invasion assay. U373MG cells exhibit the weakest invasive ability; U373MG-5 cells exhibit the strongest invasive activity. In particular, highly invasive U373MG-5 cells expressed higher levels of CYP17A1 and secreted more DHEA (Figures 1d–f) in parallel with the development of TMZ resistance (Figure 1g). To confirm whether TMZ resistance is caused by MGMT expression, we estimated MGMT expression in our established TMZ-resistant cell lines. As shown in Supplementary Figure S4, MGMT was not expressed in both U87MG-R and pt#11-R, indicating that TMZ resistance in these two cell lines is caused by MGMT-independent pathway. Furthermore, in addition to enhancing TMZ cytotoxicity in U87MG cells (Supplementary Figure S5), CYP17A1 knockdown abolished the acquired resistance of U87MG-R cells to TMZ and enhanced TMZ-induced apoptosis, as determined by caspase 3 cleavage (Figure 1h). To further investigate whether inhibition of CYP17 promotes TMZ-induced apoptosis, we used abiraterone to inhibit CYP17A1 activity. Due to the inhibition, the level of CYP17A1 was increased in a compensatory pathway (Figure 1i).^22, 23^ DHEA was significantly decreased by 20 μm of abiraterone (Figure 1i), indicating that abiraterone potently inhibits CYP17A1 activity. Therefore, under 20 μm of abiraterone treatment, we evaluated whether CYP17A1 inhibition sensitizes GBM cells to TMZ. Pretreatment with abiraterone obviously enhanced TMZ-induced apoptosis in U87MG and pt#11 cells, evidenced by poly (ADP-ribose) polymerase (PARP) and caspase 3 cleavage (Figure 1j). This finding indicates that CYP17A1 attenuates TMZ-induced apoptosis, thus leading to glioma resistance to TMZ, and suggests that CYP17A1 participates in the malignancy process of glioma. However, the mechanism responsible for CYP17A1 upregulation in GBM remains to be elucidated.

### Sp1 increases CYP17A1 transcription in glioma

**Figure 2:**
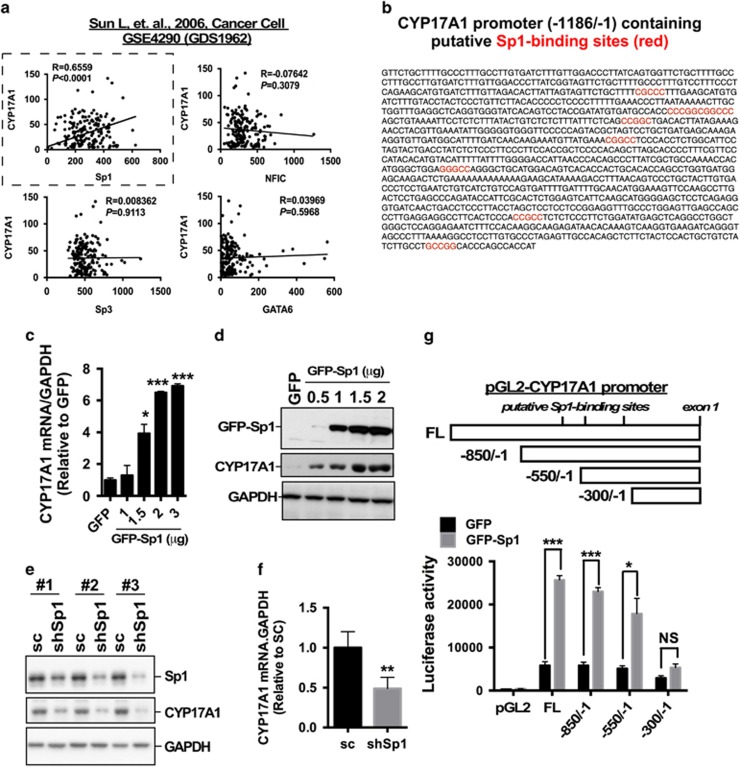
Sp1 regulates CYP17A1 expression. (**a**) The correlation of Sp1, NFIC, Sp3 and GATA6 mRNA with CYP17A1 mRNA in glioma was analyzed in the GSE4290 database. The number in x- and y-axes represent the expression value of gene in complementary DNA microarray. (**b**) The CYP17A1 promoter sequence and putative Sp1-binding sites are indicated in red. (**c**, **d**) After Sp1 overexpression or knockdown (**e**, **f**) U87MG cells were collected and subjected to western blotting with an anti-CYP17A1 antibody (left panel) and reverse transcription-quantitative PCR using primers against CYP17A1 (right panel). Sc: cells were infected by lentiviruses expressing scramble short hairpin RNA for control group; shSp1: cells were infected by lentiviruses expressing short hairpin RNA targeting Sp1. (**g**) Effects of Sp1 on different truncated promoters of CYP17A1. After transfection with the indicated plasmids for 24 hrs, the protein lysates were collected and subjected to reporter assays. Data are expressed as the means±s.e.m. (*P<0.05, ***P<0.001). NS, not significant.

As shown in Figure 1b, the upregulation of CYP17A1 mRNA correlated with glioma formation and prognosis. To determine the transcriptional mechanism underlying the aberrant CYP17A1 upregulation is our next mission. Previously, NFIC, Sp1, Sp3, GATA-4 and -6 were shown to regulate CYP17A1 expression.^24^ In particular, GATA-4 and GATA6 have been reported as a tumor suppressor gene, and as a negative regulator of astrocytoma.^25, 26^ Therefore, we excluded GATA-4 and GATA6 initially. To identify which transcription factor is a potent regulator for CYP17A1 transcription in glioma, we analyzed the correlation of CYP17A1 with NFIC, Sp1, Sp3 or GATA6 in complementary DNA microarray database of glioma (Figure 2a). We found that, neither NFIC, Sp3 nor GATA6, only Sp1 highly correlates with CYP17A1 expression. Therefore, we focus on Sp1 for further investigation (Figure 2a). In addition, we analyzed the promoter sequence of CYP17A1 and identified the presence of several GC-rich Sp1-binding sites (Figure 2b). Therefore, we investigated whether Sp1 affects CYP17A1 expression. Sp1 overexpression significantly increased both the CYP17A1 mRNA and protein levels (Figures 2c and d), whereas Sp1 knockdown significantly decreased CYP17A1 expression (Figures 2e and f). To further confirm whether Sp1 affects CYP17A1 promoter activation, the activity of the CYP17A1 promoter (−1186/−1), which had been truncated to three fragments, was analyzed in luciferase assays. As shown in Figure 2g, fragments of −850/−1 and −550/−1 exhibited the similar activity compared to full length of promoter; the CYP17A1 promoter activity was significantly decreased in the −300/−1 fragment; the positive effect of Sp1 was also abolished in this fragment but not in the −850/−1 and −550/−1 regions, thus suggesting that Sp1-regulated transcription in the −550/−300 region is important for CYP17A1 expression.

### Upregulation of Sp1-binding activity targeting the CYP17A1 promoter in TMZ resistance

**Figure 3:**
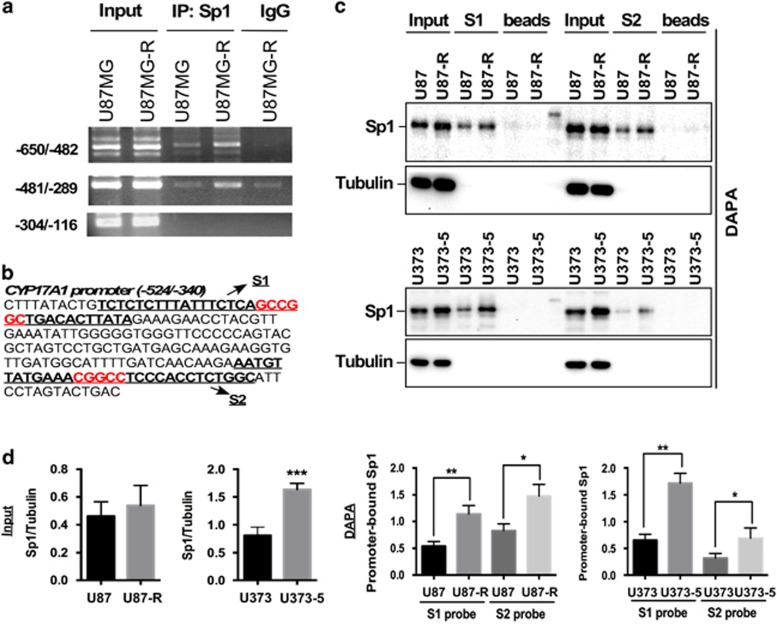
Increased Sp1-binding to the CYP17A1 promoter in TMZ-resistant glioma cells. (**a**) Cells were subjected to CHIP assays, and the indicated promoter fragment was amplified by PCR. (**b**) The CYP17A1 promoter sequence contains two putative Sp1-binding sites. (**c**) Sp1-binding activity was determined by DAPA. After incubation of protein lysates with the biotinylated Sp1-binding sequence (S1 or S2 probe) and streptavidin beads, the beads were analyzed by western blotting with an anti-Sp1 antibody. (**d**) Quantitative results for Sp1 expression and Sp1-binding activity. Data are expressed as the means±s.e.m. (*P<0.05, **P<0.01, ***P<0.001). CHIP, chromatin immunoprecipitation; DAPA, DNA affinity precipitation assay.

We then studied the mechanism of upregulation of CYP17A1 by Sp1, and found that Sp1 binds to the CYP17A1 promoter, which contains two putative Sp1-binding sites located in the −524/−340 region. This binding was further enhanced in TMZ-resistant cells evidenced by chromatin immunoprecipitation assay (Figure 3a). In addition, the binding of Sp1 to the −340/−524 region (Figure 3b) was confirmed by DNA affinity precipitation assay. In Supplementary Figure S6, Sp1 was obviously precipitated by GC-rich S1 probe, not by the negative control (non GC-rich) probe. We found that in both TMZ-resistant U87MG-R and U373MG-5 cells, the Sp1-binding activity was significantly increased (Figures 3c and d), thus suggesting that the increased Sp1-binding activity at the CYP17A1 promoter promotes CYP17A1 transcription and induces TMZ resistance.

### Sp1 competes with DNMT3a-mediated DNA methylation, thus inducing CYP17A1 expression in TMZ resistance

**Figure 4:**
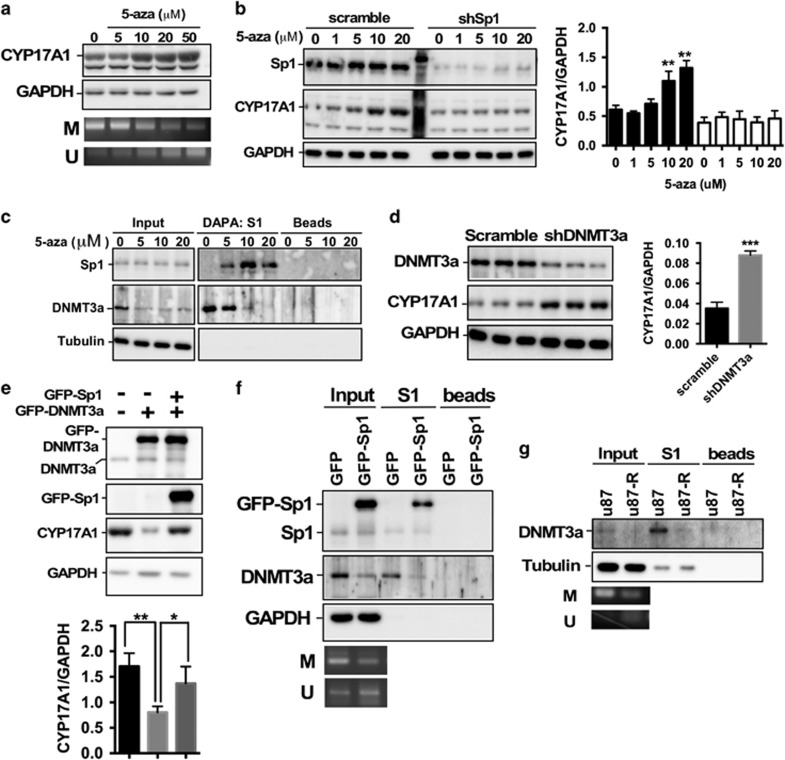
Sp1 attenuates DNMT3a-mediated DNA methylation and increases CYP17A1 expression in TMZ-resistant cells. (**a**) After treatment with 5-aza-cytidine for 24 h, the cells were collected and subjected to western blotting and MSP. (**b**) In the presence or absence of Sp1 knockdown, the cells were treated with 5-aza-cytidine for 24 h and subjected to western blotting. The quantitative result for CYP17A1 (**P<0.01) is shown in the right panel. (**c**) After treatment, the protein lysates were prepared and subjected to DAPA using the S1 probe as described in ‘Materials and Methods’. Probe-binding beads were analyzed by western blotting. (**d**) Effect of DNMT3a knockdown on CYP17A1 expression. The quantitative results for CYP17A1 (***P<0.001) are shown in the right panel. (**e**) Effects of GFP-DNMT3a overexpression on CYP17A1 expression in the absence or presence of GFP-Sp1 overexpression. Quantitative results for CYP17A1 (*P<0.05, **P<0.01) are shown in the lower panel. (**f**) After GFP-Sp1 overexpression, the binding activities of Sp1 and DNMT3a on the CYP17A1 promoter were analyzed with DAPA. (**g**) DNA-binding activities of DNMT3a in U87MG and U87MG-R were analyzed with DAPA. DAPA, DNA affinity precipitation assay; MSP, methylation-specific PCR.

Because aberrant DNA methylation has frequently been identified in recurrent GBM,^27^ we treated U87MG cells with the DNA methylation inhibitor 5-aza-cytidine and found that CYP17A1 expression was markedly increased after the inhibition of DNA methylation (Figure 4a). On the basis of this result and the previous observation that DNA methylation attenuates Sp1-binding activity,^28, 29^ we attempted to elucidate whether Sp1 regulates CYP17A1 by affecting DNA methylation. In particular, Sp1 knockdown prevented 5-aza-cytidine-induced CYP17A1 upregulation (Figure 4b) in U87MG cells, thus suggesting that CYP17A1 expression requires Sp1-mediated transcription in the absence of DNA methylation. Moreover, Sp1-binding activity to the CYP17A1 promoter was clearly increased by 5-aza-cytidine, whereas the DNMT3a-binding activity was markedly decreased (Figure 4c). However, no interactions of the CYP17A1 promoter with DNMT1 and DNMT2 were identified (Supplementary Figure S7). These results indicate that after the loss of DNMT3a-mediated methylation, the effect of Sp1 on CYP17A1 gradually increases. We next confirmed that in contrast to Sp1, DNMT3a negatively regulated CYP17A1 expression (Figures 4d and e) and that Sp1 rescued DNMT3a-mediated CYP17A1 downregulation (Figure 4e). In contrast to Sp1 knockdown, which increased methylation (Supplementary Figure S8), Sp1 overexpression abolished DNMT3a binding to the CYP17A1 promoter, thereby leading to the loss of DNA methylation (Figure 4f). This result suggests that Sp1 competes with DNMT3a in regulation of CYP17A1 expression. Importantly, DNMT3a-binding activity and DNA methylation were clearly decreased in TMZ-resistant U87MG-R cells (Figure 4g). Together, these results indicate that Sp1 attenuates DNMT3a-mediated methylation and consequently regulates CYP17A1 expression in the development of TMZ resistance.

### Sp1 controls CYP17A1-mediated DHEA secretion and promotes GBM malignancy

**Figure 5:**
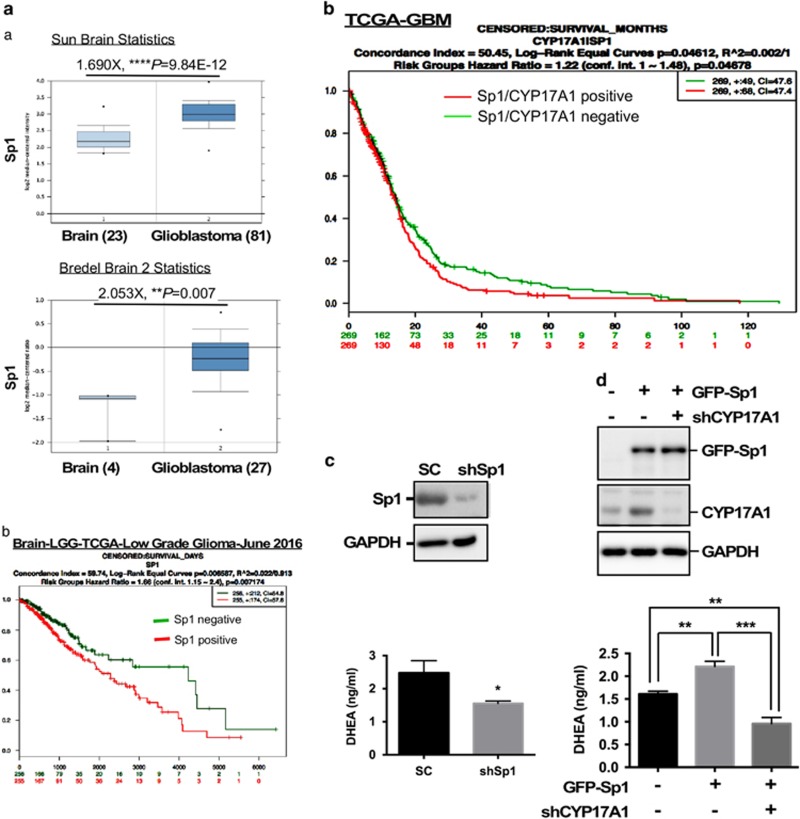
Sp1 increases DHEA production by CYP17A1 overexpression in GBM (**a**). Sp1 mRNA expression was analyzed by the oncomine website (**a**) and the survival comparison was performed by the SurvExpress website (**b**). (**b**) A survival comparison between Sp1^hi^CYP17A1^hi^ and Sp1^lo^CYP17A1^lo^ in glioma patients from the TCGA database was performed using the SurvExpress website. (**c**) After Sp1 knockdown, DHEA in the culture medium of U87MG cells was measured. (**d**) After GFP-Sp1 overexpression combined with CYP17A1 knockdown, the medium was subjected to a DHEA ELISA (lower panel), and the cells were analyzed by western blotting. Experiments were performed independently three times, and the data are expressed as the means±s.e.m. (*P<0.05, **P<0.01, ***P<0.001). ELISA, enzyme-linked immunosorbent assay.

After dissecting the mechanism by which Sp1 regulates CYP17A1 expression, we sought to highlight the importance of the Sp1–CYP17A1 axis in GBM. To elucidate whether Sp1-mediated CYP17A1 expression is involved in glioma development, we showed that Sp1 expression markedly increased as astrocytoma progressed to grade IV GBM (Supplementary Figure S9 and Supplementary Table S2) and that Sp1 strongly promoted glioma invasion (Supplementary Figures S10A–C). In particular, GFP-Sp1 overexpression increased endogenous Sp1 expression through autoregulation mechanism.^30^ Sp1 mRNA expression was also increased and significantly correlated with poor prognosis in glioma patients (Figure 5a). Interestingly, CYP17A1 knockdown abolished Sp1-enhanced invasiveness (Supplementary Figure S10D), thus further suggesting that Sp1-mediated CYP17A1 expression is required for glioma malignancy. In addition, CYP17A1 expression was markedly increased in U373MG-5 cells that exhibit higher invasiveness and Sp1 expression (Figure 1d and Supplementary Figure S10E). These results indicate that Sp1 promotes GBM development by increasing CYP17A1 expression. In addition, both Sp1 and CYP17A1 positivity significantly correlated with the prognosis of GBM patients in the TCGA database, thus further supporting the oncogenic role of the Sp1–CYP17A1 axis in GBM (Figure 5b). Consequently, Sp1 knockdown significantly decreased DHEA secretion (Figure 5c), and CYP17A1 knockdown significantly abolished Sp1-induced DHEA secretion (Figure 5d). These results suggest that the overactivation of the Sp1–CYP17A1 axis may be responsible for DHEA overproduction, leading to GBM malignancy.

### DHEA attenuates TMZ-mediated apoptosis

**Figure 6:**
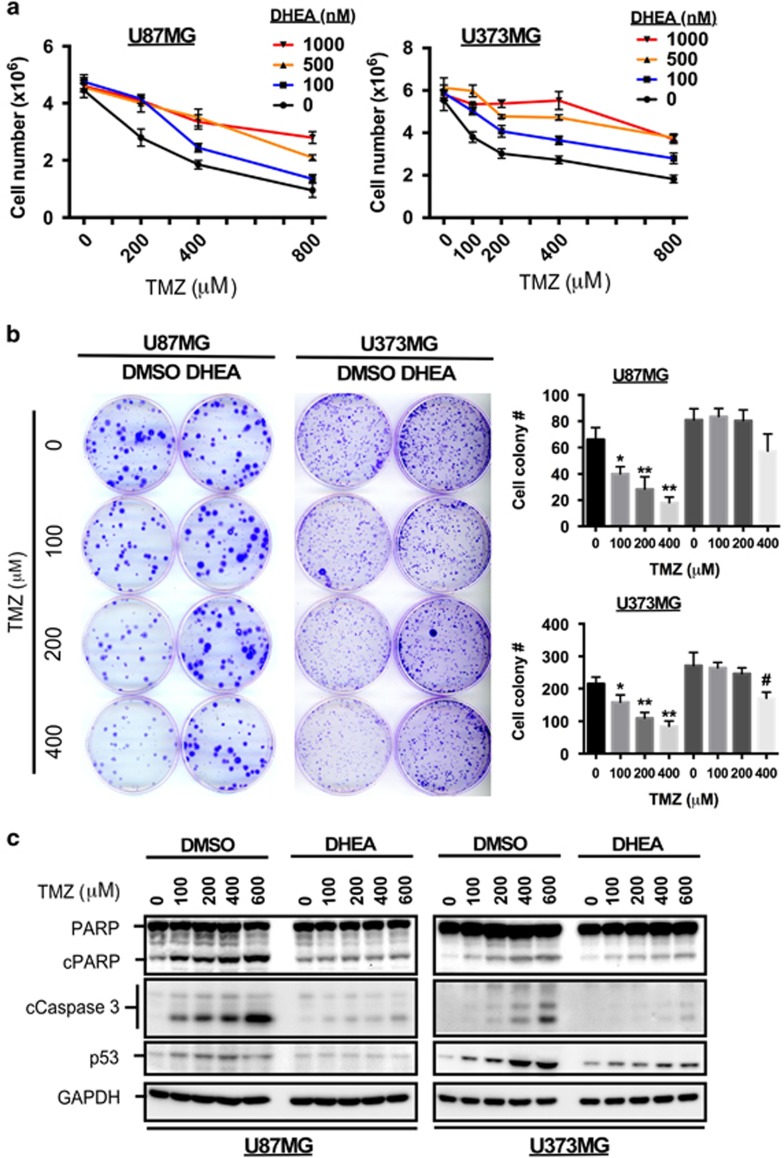
DHEA attenuates TMZ-induced apoptosis. (**a**) After treatment with DHEA for 48 h, the cells were treated with TMZ for an additional 48 h in the absence of DHEA. The cells were collected for cell counting with a hemacytometer. (**b**) Cells pretreated with 1 μm of DHEA for 4 days were treated with TMZ for an additional 8 days. Crystal violet was used to stain the cells. Quantitative results for CYP17A1 (*P<0.05, **P<0.01 indicate the difference between the groups with or without TMZ treatment; ^#^*P*<0.05 indicates the difference between TMZ-treated U373MG and DHEA+TMZ-treated U373MG cells) are shown in the right panel. (**c**) After treatment with DHEA for 48 h, the cells were treated with TMZ for an additional 48 h in the absence of DHEA. Protein lysates were subjected to western blotting with anti-PARP, anti-caspase 3 and anti-p53 antibodies.

**Figure 7:**
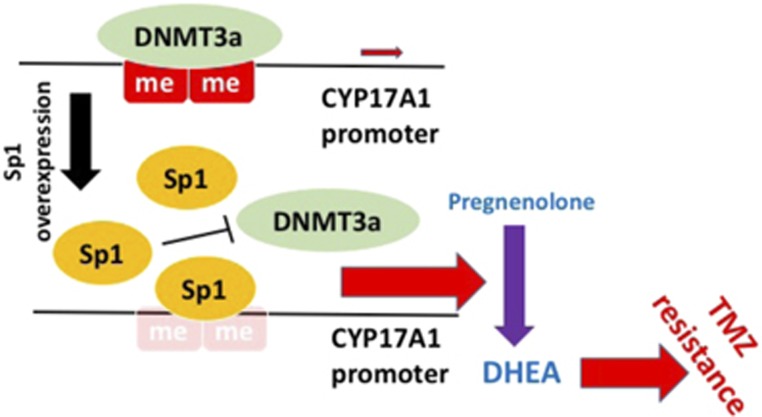
Sp1 induces CYP17A1 overexpression by inhibiting DNMT3a-mediated DNA methylation, thus resulting in an increase in DHEA biosynthesis and glioma resistance to TMZ.

The major function of CYP17A1, which is markedly increased in TMZ-resistant cells, is the conversion of pregnenolone to DHEA.^1^ DHEA has also been shown to protect against neuronal cell death under oxidative stress.^2, 31^ These findings prompted us to determine whether DHEA protects GBM cells from TMZ-induced apoptosis, thus leading to resistance. As shown in Figure 6, the inhibitory effect of TMZ on glioma cell proliferation was gradually attenuated by increasing doses of DHEA (Figure 6a). In addition, colony formation assays revealed that cells pretreated with DHEA for 4 days exhibited greater resistance to TMZ for the subsequent 8 days (Figure 6b). Furthermore, TMZ-induced apoptosis, as assessed on the basis of cleaved PARP and caspase 3 activity, was clearly abolished by DHEA pretreatment (Figure 6c). In addition, DHEA also prevented TMZ-induced DNA damage, as evidenced by increased p53 expression (Figure 6c).^32^ These results suggest that DHEA protects glioma cells from TMZ-induced apoptosis by attenuating DNA damage. These findings indicate that as GBM develops resistance to TMZ, CYP17A1 expression is increased because of the decrease in DNA methylation of the CYP17A1 promoter. In particular, Sp1 competes with DNMT3a, upregulates CYP17A1 expression and confers TMZ resistance by increasing DHEA biosynthesis in GBM (Figure 7).

## Discussion

Although Sp1 regulates tumorigenesis by affecting several cellular processes, the role of Sp1 in tumor metabolism, especially in steroidogenesis, remains poorly understood. In addition, whether CYP17A1, the key steroidogenic enzyme in DHEA biosynthesis, affects glioma malignancy, including invasion and drug resistance, is also unknown. In contrast to the evidence indicating that DHEA protects neurons from stress-induced apoptosis,^2^ the role of DHEA in glioma progression has not been reported. In the present study, we found that CYP17A1 expression was significantly increased by Sp1 and correlated with poor prognosis in glioma patients. In particular, CYP17A1 upregulation was caused by the inhibitory effect of Sp1 on DNMT3a-mediated CYP17A1 repression, and CYP17A1 induced TMZ resistance by increasing DHEA production in GBM.

The results of Sp1-regulated CYP17A1 transcription are not the same with that in human adrenal NCI-H295A cells in which the transcription of CYP17A1 gene is majorly contributed by NFIC, Sp1 and Sp3.^24^ They also identified Sp1/Sp3, and NFIC regulate CYP17A1 transcription through binding to the −227/−184 and −107/−85 regions, respectively.^24^ However, in the complementary DNA microarray database of glioma, GSE4290 in Figure 2a, Sp1 expression, neither NFIC nor Sp3, significantly correlates with CYP17A1 expression, suggesting that Sp1, not NFIC and Sp3, regulates CYP17A1 expression for glioma. In addition, we think the Sp1-regulated regions locate at the −524/−340 region based on Figures 2g and 3. This is different with the previous report indicating Sp1 regulates the −227/−184 region of CYP17A1 promoter in NCI-295A cells.^24^ In Figure 2g, Sp1-induced transcription is significantly abolished in the −300/−1 fragment, not in −850/−1, −550/−1 fragments. This indicates that Sp1-regulated region locates at the -550/-300 that contains two putative Sp1-binding sites. Furthermore, Sp1 associates with −514/−480 and −380/−355 regions evidenced by DNA affinity precipitation assay (Figure 3), indicating that Sp1 promotes CYP17A1 transcription through associating to these two regions. On the basis of our findings, Sp1-regulated regions of CYP17A1 promoter in GBM cells is different with those in adrenal NCI-H295A cells. The underlying discrepancy needs to be elucidated in the future.

Inhibition of the cholesterol transporter or internalization for cellular utilization is sufficient to induce autophagy-dependent apoptosis,^33, 34^ thus suggesting a high demand for cholesterol in gliomas. In addition to studying CYP17A1, we also evaluated whether Sp1 affects other steroidogenic enzymes, such as CYP11A1 and steroidogenic acute regulatory protein, both of which mediate the rate-limiting and the conversion of cholesterol to pregnenolone.^1^ Although these proteins are regulated by Sp1 in the adrenal gland,^35^ we did not identify their upregulation in GBM patients and did not observe any positive effects of Sp1 on these two genes (data not shown). Therefore, we focused on Sp1-mediated CYP17A1 expression in GBM. Although pregnenolone exhibits a pro-apoptotic effect in glioma,^36^ its metabolite, DHEA, synthesized by CYP17A1, contributes to TMZ resistance (Figure 6). The other metabolite that exhibits similar effects in astrocytes, allopregnanolone, promotes astrocyte proliferation.^9^ Given the neuroprotective effect of DHEA, we hypothesized that DHEA might antagonize the effects of TMZ. Previous studies have shown that TMZ induces apoptosis by inducing DNA damage, which results in the accumulation of reactive oxygen species,^32, 37^ and DHEA protects neurons from reactive oxygen species-dependent apoptosis.^2^ Several anti-oxidant enzymes that remove reactive oxygen species, such as superoxide dismutase, NADPH oxidase, peroxidase and catalase,^31, 38^ are regulated by DHEA. Therefore, DHEA decreases the reactive oxygen species levels via an unknown signaling pathway, thereby preventing apoptosis in glioma. Whether DHEA regulates drug sensitivity by acting on the androgen receptor, the G protein-coupled receptor, the Sigma-1 receptor or NMDA, all of which mediate DHEA actions in neurons, is also unknown.^39, 40, 41^ This issue still requires further elucidation.

The effect of DHEA on tumor is still controversial. In different cell types of, DHEA may exhibit different action. DHEA inhibits Leydig cell proliferation^42^ and exhibits anti-tumor effect in cervical cancer^43^ and myeloma;^44^ whereas DHEA promotes proliferation of prostate epithelial cell^45^ and vascular endothelial cells,^46^ and exhibits tumor-promoting effect on ER^+^ breast cancer cell.^47^ Moreover, DHEA also promotes proliferation of cells in the brain, including progenitor cells in the dentate gyrus^48^ and neuronal cells.^49^ The underlying discrepancy remains unclear. The tumor-promoting effect of DHEA may be caused by activation of non-genomic pro-survival signal transduction.^39^ DHEA increases the secretion of BDNF and NGF, both of which promotes proliferation of astrocytes.^49^ DHEA activates both PI3K/Akt and Src/Erk1/2 pathways that are highly activated in cancer development.^39, 50, 51^ In our study, DHEA induces TMZ resistance in GBM. The underlying mechanism may be related with activation of PI3K/Akt and Src/Erk pathway. However, this part still needs further investigation.

In Figure 6, we found that TMZ strongly increased p53 expression in U373MG, compared with U87MG cells. The underlying mechanism is not clear. Previously, several reports indicate that TMZ increases the level of p53, indicating that TMZ induces DNA damage response.^52, 53, 54^ U373MG expresses mutant p53^R273H^, which is the gain of function for p53 protein.^55^ This suggests that U373MG is more sensitive in response to DNA damage induction, compared to U87MG cells. In evaluating the protein level of p53 in U87MG, pt#11 and U373MG cells, we found that U373MG cells have higher p53 expression and p53 phosphorylation in S15 and S33 residues (Supplementary Figure S11). This may reflect that U373MG is more sensitive to TMZ treatment that potently induces DNA damage. On the basis of these, in U373MG cells, TMZ is more potent in inducing DNA damage characterized by p53 expression.

However, in both U87MG and U373MG cells, DHEA protected cells from TMZ-induced apoptosis. Therefore, we suggest that effect of DHEA on glioma cells is not affected by p53 status.

GBM progression, including invasion and acquired drug resistance, is tightly associated with DNA methylation. In gliomas, compared with normal brain tissue, promoter hypermethylation of the *RB*, *CDKN2A*, *PTEN* and *P53* genes has been clearly identified, and these changes serve as prognostic predictors.^27^ The other well-known mechanism is the methylation of the MGMT promoter, which leads to MGMT-dependent TMZ resistance and recurrence.^32^ These findings indicate that DNA methylation actively participates in regulating glioma progression. In addition, Sp1 overexpression exacerbates the prognosis of GBM patients as a result of enhanced proliferation, invasion and the reduced sensitivity of cells to chemotherapy.^19^ In spite of these findings, the interaction of Sp1 with DNA methylation in GBM has not been clearly defined. Sp1 has previously been found to bind to hypomethylated promoters, and DNA methylation inhibits Sp1-mediated transcription.^28, 29^ Similar results are shown in Figure 4c: Sp1-binding to the CYP17A1 promoter gradually increased after inhibition of DNA methylation. Interestingly, Sp1 is involved in ribosomal DNA demethylation in ribosome biogenesis,^56^ but it is unclear how Sp1 causes DNA demethylation. Here, we identified a possible mechanism. As shown in Figure 4, Sp1 competes with DNMT3a and attenuates DNA methylation of the CYP17A1 promoter. This result suggests that Sp1 increases CYP17A1 transcription, possibly through DNA demethylation rather than an Sp1-mediated transcription mechanism completely dependent on the promoter methylation status.

Results from Liu *et al.*^57^ indicating that 5-aza-cytidine decreases CYP17A1 mRNA levels are different with our study showing that 5-aza-cytidine increases CYP17A1 proteins (Figure 4). In addition to different cell types (NCI-H295A vs U87MG), treatment time is also distinct (7 vs 1 day). These two experimental conditions may cause different consequences. However, whether treatment with 5-aza-cytidine for 7 days decreases CYP17A1 in GBM needs further investigation.

In the process of glioma malignance, tumor cells become more aggressive in enhancing both migratory and invasive ability. After TMZ treatment, aggressive glioma cells acquire resistance in MGMT-dependent and -independent pathways. Some reports also indicate that tumor cells possessing stronger invasive ability have the lower sensitivity to chemotherapy. Epithelial–mesenchymal transition that highly enhances the invasiveness enriches CD44-positive cancer stem cells,^58^ and transcription factors responsible for epithelial–mesenchymal transition also induce gene expression related to chemoresistance,^59^ including ATP-binding cassette transporter, Bmi1 and Nanog.^60^ In particular, epithelial–mesenchymal transition-induced ZEB1 contributes to invasion and chemoresistance in GBM.^61, 62^ In addition, transforming growth factor β that induces cell invasion was shown to maintain tumorigenecity of GBM through maintaining stemness of cancer-initiating cells.^63^ Therefore, we attempt to evaluate the role of the Sp1–CYP17A1 axis in the enhancement of invasiveness and study whether this axis causes TMZ resistance in U373MG-series cell model. However, the mechanism underlying migration-induced TMZ resistance remains unclear.

In the past decade, owing to the important role of CYP17A1-mediated steroidogenesis in cancer development, several generations of inhibitors targeting CYP17A1 have been developed to treat prostate cancer; these inhibitors include abiraterone, orteronel, galaterone and seviteronel.^64^ In particular, abiraterone acetate has recently been approved in the United States for the treatment of hormone-dependent and castration-resistant prostate cancer patients.^65^ In addition, the inhibition of CYP17 by ketoconazole also exhibited tumor-suppressive effects in ovarian cancer.^66^ These results suggest that CYP17A1 inhibition is a potential target for the treatment of cancer patients. However, studies of tumor steroidogenesis have mainly focused on prostate cancer, and the effects of drugs targeting steroidogenic enzymes in other types of cancer have rarely been evaluated. Therefore, further investigation is required to determine whether drugs targeting CYP17A1 may be useful for the treatment of glioma patients.

## Materials and methods

### Cell culture and treatment

Glioma cell lines, including A172, U87MG and U373MG, and the astrocyte cell line SVG-P12 were purchased from ATCC (Manassas, VA, USA) and cultured in Dulbecco’s Modified Eagle’s Medium (Thermo Fisher Scientific, Waltham, MA, USA) supplemented with 10% fetal bovine serum (GE Healthcare Life Sciences, South Logan, UT, USA), 100 μg/ml penicillin and 100 μg/ml streptomycin (Thermo Fisher Scientific) at 37 °C in a 5% CO_2_ incubator. For cell counting, the cells were pretreated with DHEA (Sigma-Aldrich, St Louis, MO, USA) for 48 h and then treated with TMZ (Sigma-Aldrich) for an additional 48 h. Abiraterone was purchased from Selleckchem (Houston, TX, USA).

### Human specimens

The use of human specimens adhered to the institutional human ethics guidelines and was approved by the Clinical Research Ethics Committee at Taipei Medical University Hospital. The human plasma was provided by the Biobank of Taipei Medical University (Taipei, Taiwan). The human GBM cell line pt#11 was isolated from the freshly excised tumor tissue of a male patient affected by a grade IV GBM. After homogenizing by collagenase IV and DNase I, cells of glioma tissue were seeded onto six-well plates and cultured in Dulbecco’s Modified Eagle’s Medium supplemented with 10% fetal bovine serum. A human tissue array was purchased from US Biomax, Inc. (Rockville, MD, USA). The lysate of human normal brain tissue was purchased from Abcam (Cambridge, MA, USA).

### Bioinformatics

Expression of CYP17A1 and Sp1 in tumor and normal brain tissues was analyzed using the Oncomine website (https://www.oncomine.org/resource/login.html). To investigate the correlation of Sp1 expression with CYP17A1, expression value of complementary DNA microarray in GSE4290-GDS1962 of NCBI was analyzed by linear regression. To determine the prognosis of glioma patients, SurvExpress was used according to the website instructions.^67^ The PROMO websites (http://alggen.lsi.upc.es/cgi-bin/promo_v3/promo/promoinit.cgi?dirDB=TF_8.3) was used to predict Sp1-binding sites. CYP17A1 reference sequence: NM_000102.3 (https://www.ncbi.nlm.nih.gov/nuccore/NM_000102). The 1186 bp before exon 1 was defined as the promoter.

### Enzyme-linked immunosorbent assay for DHEA

To detect DHEA in human plasma and culture media, an enzyme-linked immunosorbent assay kit for DHEA was purchased from Enzo Life Sciences, Inc. (ADI-900-093; Farmingdale, NY, USA) and was used according to the manufacturer’s instructions.

### DNA affinity precipitation assay

Probes containing the putative Sp1-binding site of the CYP17A1 promoter were synthesized and biotinylated at the 5′ terminus by Genomics BioSci & Tech (New Taipei City, Taiwan) as follows: S1: biotin-TCTCTCTTTATTTCTCAGCCGGCTGACACTTATA; S2: biotin-AATGTTATGAAACGGCCTCCCACCTCTGGC. After annealing of probe complementary strands, protein lysates were incubated with 1 μm probe in 500 μl of binding buffer containing 2 mm MgCl_2_, 60 mm KCl, 20 mm Tris-HCl, pH 7.8, 5% glycerol, 0.5 mm EDTA and 0.02% NP-40 for 1 h at 4 °C with rotation. Subsequently, the mixture was incubated with 40 μl streptavidin agarose beads (Sigma-Aldrich) at 4 °C for 1 h. Finally, the beads were washed with radioimmunoprecipitation assay buffer (10 mm Tris-HCl, pH 7.4, 150 mm NaCl, 5 mm EDTA, 0.1% sodium dodecyl sulfate, 1% Nonidet P-40 and 0.25% deoxycholate) and the bound proteins were eluted in 2 × sample buffer (3.33% sodium dodecyl sulfate, 116.67 mm Tris-HCl, pH 6.8, 10% glycerol, 0.004% bromophenol blue and 1% β-mercaptoethanol) for western blotting.

### Chromatin immunoprecipitation assay

Briefly, DNA precipitated by using the anti-Sp1 antibody was amplified by PCR using primers targeting the CYP17A1 promoter as follows: −650/−482, F: TGTTCTTACACCCCCTCCCCTTTT, R: TATAAGTGTCAGCCGGCTGAGAA; −81/−289, F: GAAAGAACCTACGTTGAAATATT, R: ACGAAAGGGGTGCTAAGCTGTG; −304/−116: F: CTTCCACCGCTCCCCACAGCT, R: TGTTATCTCTTGCCTTGTGGA.

### Methylation-specific PCR

To analyze CYP17A1 promoter methylation, genomic DNA was extracted using a QIAamp DNA Mini Kit (Qiagen, Inc., Valencia, CA, USA). After DNA bisulfite conversion using an EZ DNA Methylation-Gold Kit (Zymo Research Corp., Irvine, CA, USA), DNA was subjected to methylation-specific PCR. The primers for methylation-specific PCR were designed according to the Li laboratory website (Peking Union Medical College Hospital, Chinese Academy of Medical Sciences) and synthesized by Genomics BioSci & Tech as follows: F-TGGTTGATATTTATAGAAAGAATTTATGT; R-AAAATACCAAAAATAAAAAACCATT. The PCR products were analyzed by 2% agarose gel electrophoresis in the presence of ethidium bromide (Sigma-Aldrich).

### Statistical analysis

Differences between the two groups were analyzed by using Student’s *t*-tests. The survival analysis was performed by using the Kaplan–Meier method, and the survival rates of the two groups were compared by using log-rank tests. *P*<0.05 was considered to indicate a statistically significant difference.
