# Supplementary material for: Upregulation of CYP17A1 by Sp1-mediated DNA demethylation confers temozolomide resistance through DHEA-mediated protection in glioma
Source: Oncogenesis. 2017 May 22;6(5):e339–. doi: 10.1038/oncsis.2017.31 (PMC5523064; doi:10.1038/oncsis.2017.31)
Supplement: Supplementary Information [file oncsis201731x1.docx]

**Supplementary materials**

**Upregulation of CYP17A1 by Sp1-mediated DNA Demethylation Confers Temozolomide Resistance through DHEA-Mediated Protection in Glioma**

Jian-Ying Chuang^#^, Wei-Lun Lo^#^, Chiung-Yuan Ko, Szu-Yi Chou, Ruei-Ming Chen, Kwang-Yu Chang, Jan-Jong Hung, Wu-Chou Su, Wen-Chang Chang^*^, Tsung-I Hsu^*^

Supplementary Materials and Methods

^Supplementary Figures: 11^

^Supplementary Table: 2^

**Supplementary Materials and Methods**

**Establishment of TMZ-resistant cells**

After treatment with 100 μM TMZ for 24 hrs, the cells were seeded into 96-well plates (1 cell/well), on Day 0. During Days 1 to 21, cells were incubated with growth media containing 100 μM TMZ; days 22-42, the dose of TMZ was increased to 200 μM; days 43-63, the dose of TMZ was increased to 300 μM. In this process, the media containing TMZ was renewed once/2 days. The survived cells in the individual well in the 96-well plate were expanded into 24-well plates. Subsequently, health cells were gradually expanded into 12-well plates, 6-cm and 10-cm dishes in the presence of 300 μM TMZ. Finally, TMZ-resistant cells were maintained in the presence of 300 μM TMZ. The sensitivity to TMZ was tested by using a colony formation assay.

**In vitro matrigel-combined invasion assay**

24-well Transwell plates with 8-μm pore size polycarbonate filter membranes (Corning Costar, Corning, NY, USA) were used. The details of this experiment were as described in a previous study ^1^. For selection of highly invasive cells, ten thousand of cells were seeded onto membrane of inserted chamber and incubated for 24 h. The cells, which traverse the membrane and attach on the bottom field, were expanded. For U373MG-1 cells, ten thousand of U373MG cells were seeded onto membrane of inserted chamber and incubated for 24 h. The traversed cells were incubated and named as U373MG-1; For U373MG-2 cells, ten thousand of U373MG-1 cells were seeded onto membrane of inserted chamber and incubated for 24 h. The traversed cells were incubated and named as U373MG-2. For U373MG-3 cells, ten thousand of U373MG-2 cells were seeded onto membrane of inserted chamber and incubated for 24 h. The traversed cells were incubated and named as U373MG-3. For U373MG-4 cells, ten thousand of U373MG-3 cells were seeded onto membrane of inserted chamber and incubated for 24 h. The traversed cells were incubated and named as U373MG-4. For U373MG-5 cells, ten thousand of U373MG-4 cells were seeded onto membrane of inserted chamber and incubated for 24 h. The traversed cells were incubated and named as U373MG-5. The invasive activity of U373MG-series cell lines was tested by invasion assay.

**Immunohistochemistry (IHC)**

IHC was performed using a VECTASTAIN Elite ABC HRP kit (Vector Laboratories, Inc., Burlingame, CA, USA) according to the manufacturer’s instructions, as described in a previous study ^1^.

**Reporter assay**

After cells were transfected with the indicated plasmids for 24 hrs, the cell lysates were harvested in Luciferase Cell Culture Lysis Reagent (Promega Corporation, Madison, WI, USA) and used in luciferase reporter assays. Luminescence activity was measured by using a luminometer (Hidex, Turku, Finland).

**RT-qPCR**

RNA was extracted using Trisure reagent (SignaGen Laboratories) according to the manufacturer’s instructions. After reverse transcription with a SensiFAST^TM^ cDNA Synthesis Kit (Bioline USA, Inc., Taunton, CA, USA), 1 ng of cDNA was subjected to real-time PCR with a SensiFAST™ SYBR® Hi-ROX Kit (Bioline USA Inc.) in a StepOnePlus^TM^ Real-Time PCR System (Thermo Fisher Scientific). Primers for qPCR are: CYP17A1, F- ATCATAGACAACCTGAGCAAA, R- TCAGCAGATCATTTCGTA TTT.

**Plasmids**

The 1186 bp of CYP17A1 promoter (-1186/-1) was amplified in genomic DNA of U87MG cells by using primers. Full length (-1186/-1): F: 5’- GCTAGCGTTCTGCTTTTGCCCTTT-3’, R: 5’- CCGGCACCCAGCCACCATCTCGAG-3’. -850/-1: F: 5’- GCTAGCTGTAAAATTCCTCTCTTTA-3’, R: 5’- CCGGCACCCAGCCACCATCTCGAG-3’. -550/-1: F: 5’- GCTAGCTTATCGCTGCCAAAACCACA-3’, R: 5’- CCGGCACCCAGCCACCATCTCGAG-3’. -300/-1: F: 5’- GCTAGCGCATGGGGAGCTCCTCAGAG-3’, R: 5’- CCGGCACCCAGCCACCATCTCGAG-3’. The forward primers contain NheI restriction enzyme sites; the reverse primer contains XhoI enzyme site. The PCR product was purified from ethidium bromide-stained agarose gel, and ligated into pGL2 vector. GFP-Sp1 represents pEGFP-Sp1 which was constructed and described previously ^2, 3^.

**Lentivirus-mediated gene knockdown**

shRNA-expressed lentiviruses targeting scramble (ASN0000000004), Sp1 (NM138473; TRCN0000274208) or CYP17A1 (NM000102; TRCN0000064303) were constructed by the RNAi Core Facility of Academic Sinica (Taipei, Taiwan) and were used according to the provider’s instructions.

**Transfection**

Cells were transfected with the indicated plasmids with Polyjet reagent (SignaGen Laboratories, Rockville, MD, USA) according to the manufacturer’s instructions.

**Western blotting**

Briefly, blocked polyvinylidene difluoride membranes were incubated with primary antibodies, such as anti-Sp1 (EMD Millipore, Billerica, MA, USA), CYP17A1 (Proteintech Group, Chicago, IL, USA), anti-DNMT3a (Abcam, Cambridge, MA, USA), anti-DNMT1 (Santa Cruz Biotechnology, Inc., Dallas, Texas, USA), anti-DNMT2 (Santa Cruz Biotechnology, Inc.), anti-PARP (GeneTex, Inc., Irvine, CA, USA), anti-MGMT (Santa Cruz Biotechnology, Inc.), anti-caspase 3 (Cell Signaling Technology, Danvers, MA, USA), anti-p53 (Cell Signaling Technology), anti-p-p53-S15 (Cell Signaling Technology), anti-p-p53-S33 (Cell Signaling Technology), anti-tubulin (Sigma-Aldrich), or anti-GAPDH (Proteintech Group) overnight at 4ºC and then incubated with horseradish peroxidase (HRP)-conjugated anti-rabbit or anti-mouse antibodies for 1 hr at room temperature. Detection signals amplified with Enhanced Chemiluminescence Reagent (ECL, GE Healthcare Life Sciences) were captured by using a ChemiDoc^™^ Touch Imaging System (Bio-Rad Laboratories, Inc., Hercules, CA, USA).

**Colony formation assay**

To confirm the resistance of glioma cells in response to TMZ, one hundred U87MG and U87MG-R cells, five hundred pt#11 and pt#11-R cells and one thousand U373MG and U373MG-5 cells were seeded onto 6-cm dishes. After incubation for 3 days, the cells were treated with different doses of TMZ and then incubated for 9 additional days. To evaluate the effect of DHEA on TMZ-induced cell death, cells pre-treated with DHEA for 4 days were treated with TMZ for 8 additional days. The cell colonies were stained with 0.05% crystal violet (Sigma-Aldrich) overnight.


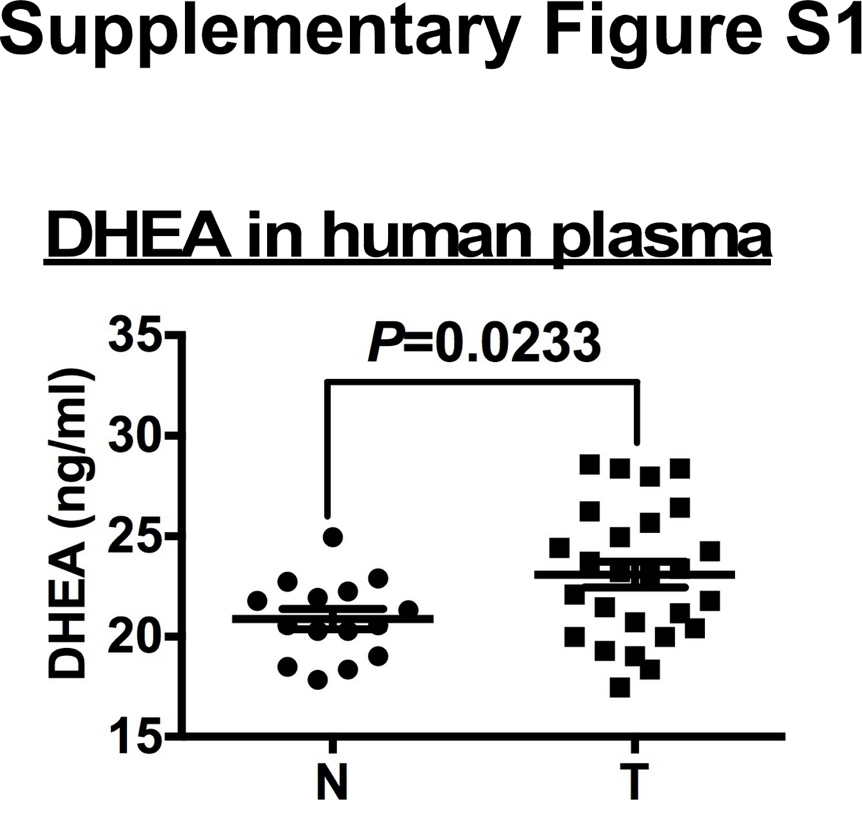


**Supplementary Figure S1. DHEA in human plasma including normal and glioma patients.** The plasma of patients (26) and non-cancer individuals (15) was collected and stored at -80 until the analysis by ELISA.


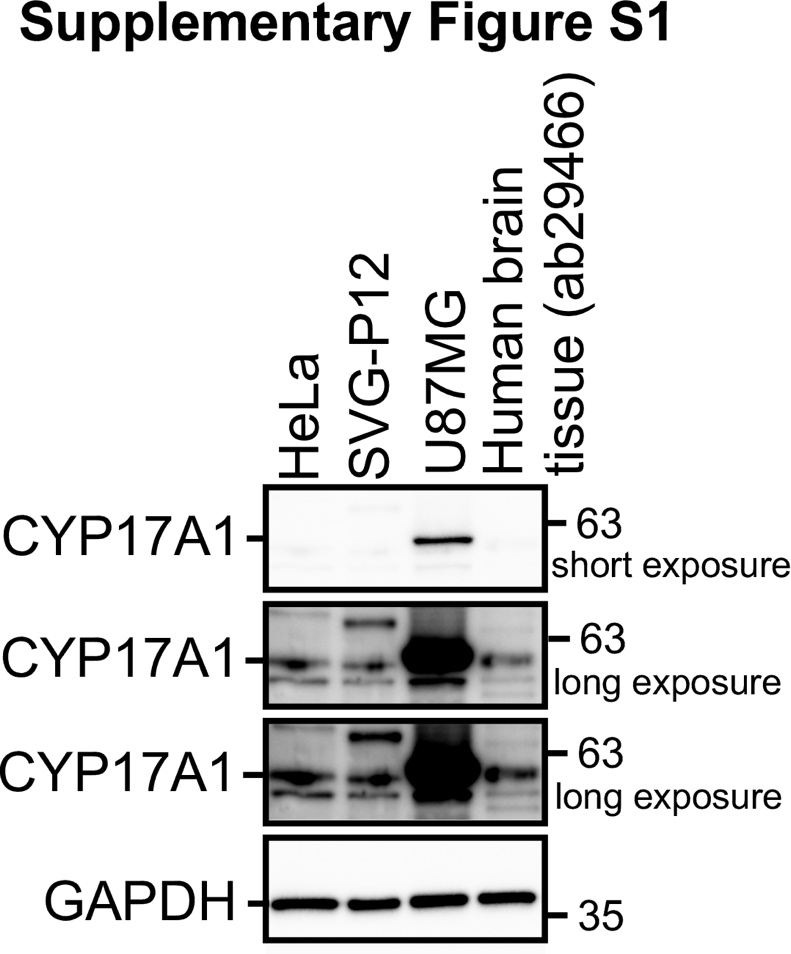


**Supplementary Figure S2. CYP17A1 expression in human brain tissue.** The lysates of indicated cells were harvested, and the lysate of human normal brain tissue was purchased from abcam (Cat. No. ab29466). The protein level of CYP17A1 was detected by wester blotting.


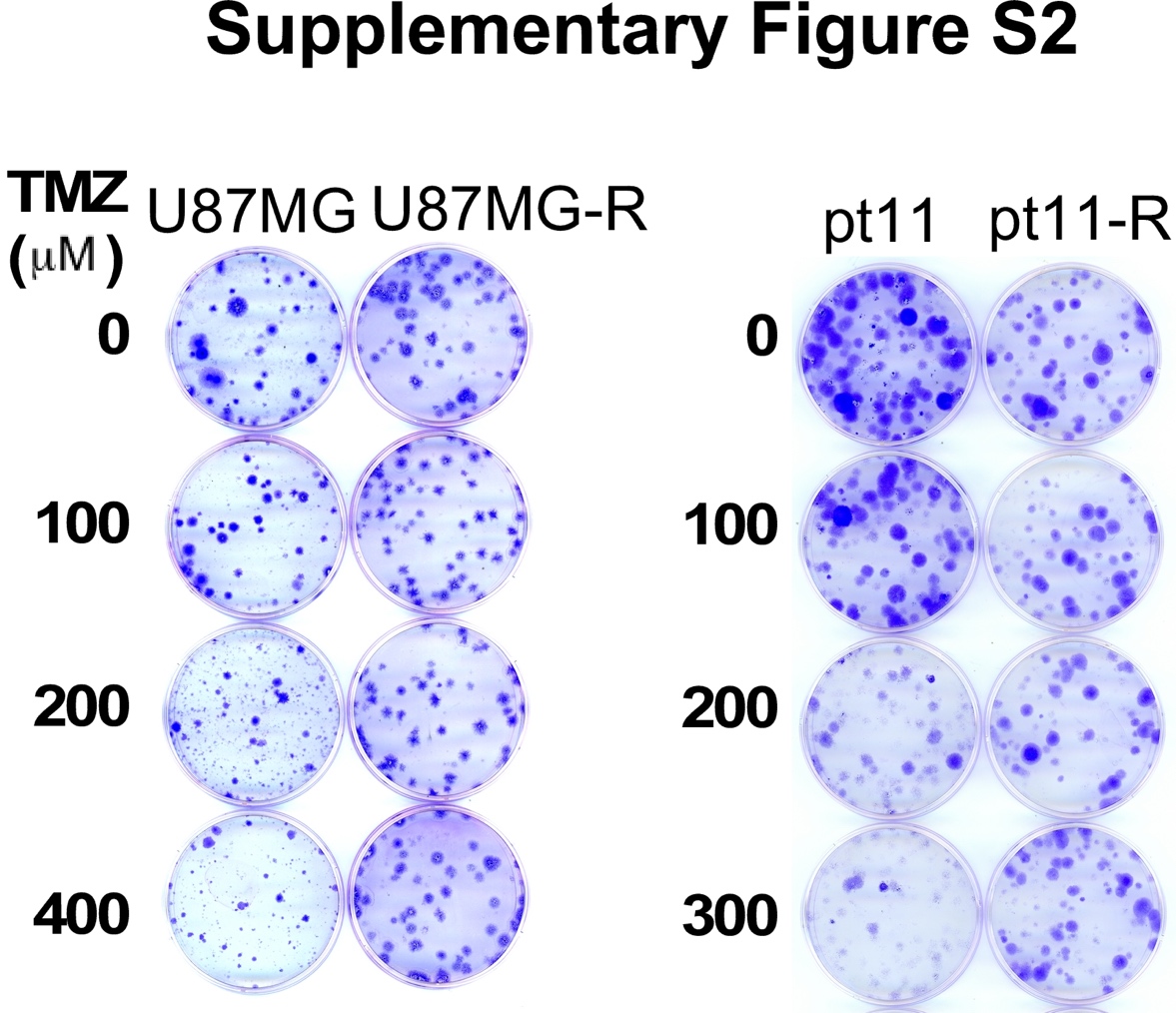


**Supplementary Figure S3. To confirm the resistance of established cell lines, colony formation assay was performed.** After seeding cells for 3 days, cells were treated with TMZ for 9 days and stained by crystal violet.


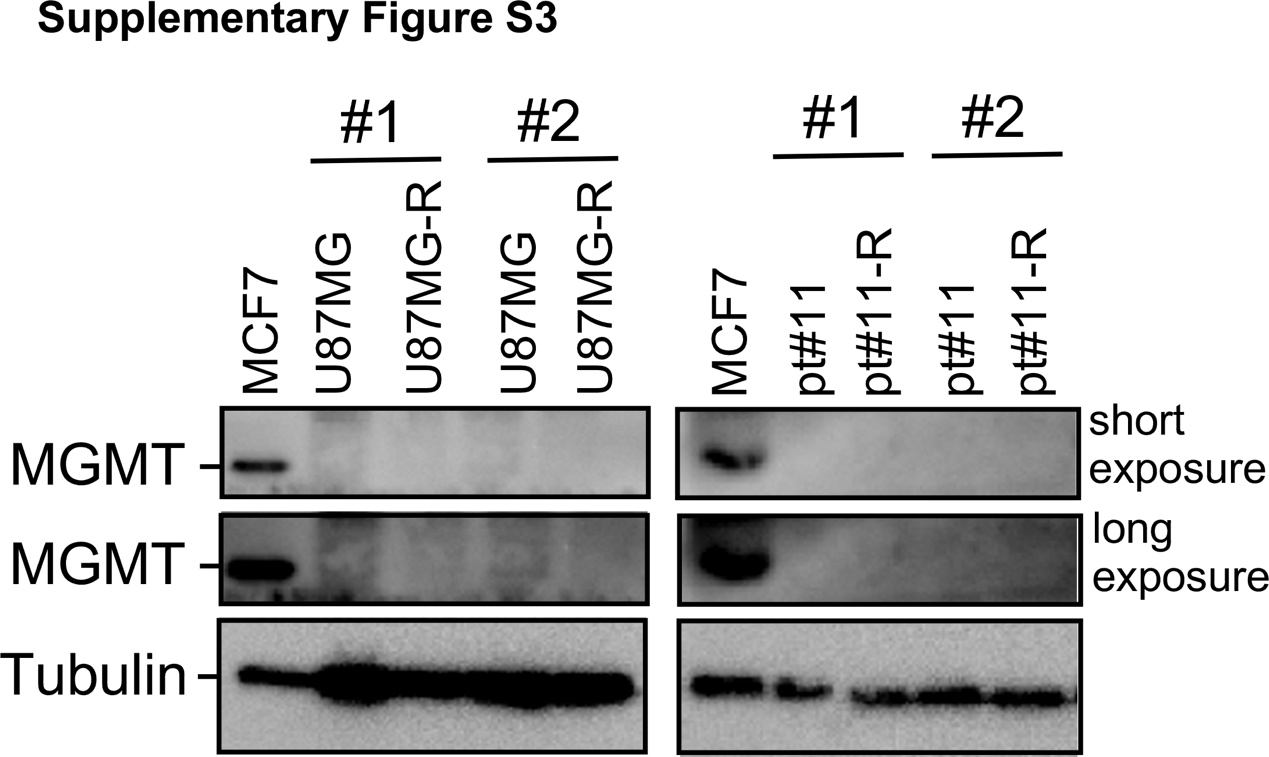


**Supplementary Figure S4. MGMT expression in GBM cell lines with or without TMZ resistance.** The lysates were collected for western blotting using the anti-MGMT antibody. The MCF7 was a positive control.


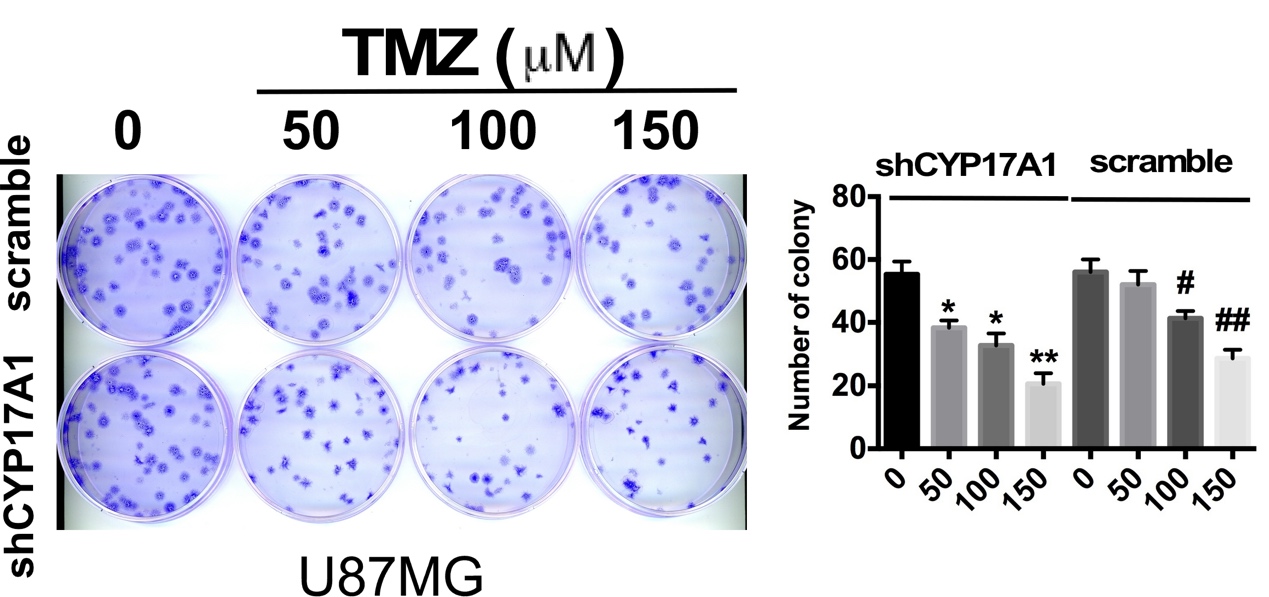


**Supplementary Figure S5. Effect of CYP17A1 knockdown on TMZ-mediated cytotoxicity in U87MG cells.** Left panel: After CYP17A1 knockdown, a colony formation assay (left panel) was performed and quantified (right panel). Data are expressed as the means±s.e.m. (***P*<0.01 indicates the difference between the shCYP17A1-group with or without TMZ treatment; ^##^*P*<0.01 indicates the difference between the scramble-group with or without TMZ treatment).


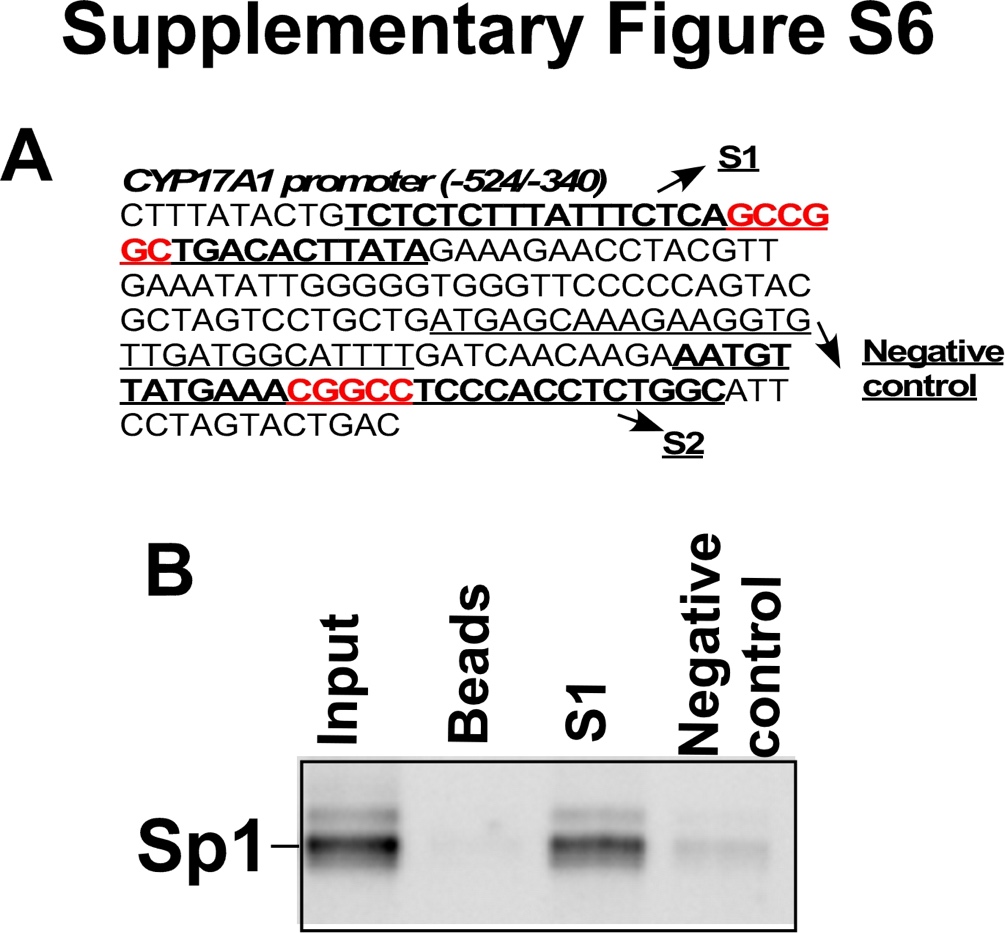


**Supplementary Figure 6. DAPA assay for evaluating the binding of Sp1 with the GC-rich probe.** A. The biotin-labeled sequences (the same with Figure 3B). B. After incubation of U87MG protein lysates with the biotinylated Sp1-binding sequence (S1 probe) and the negative control probe, streptavidin-precipitated protein-probe complex was analyzed by western blotting using the anti-Sp1 antibody.


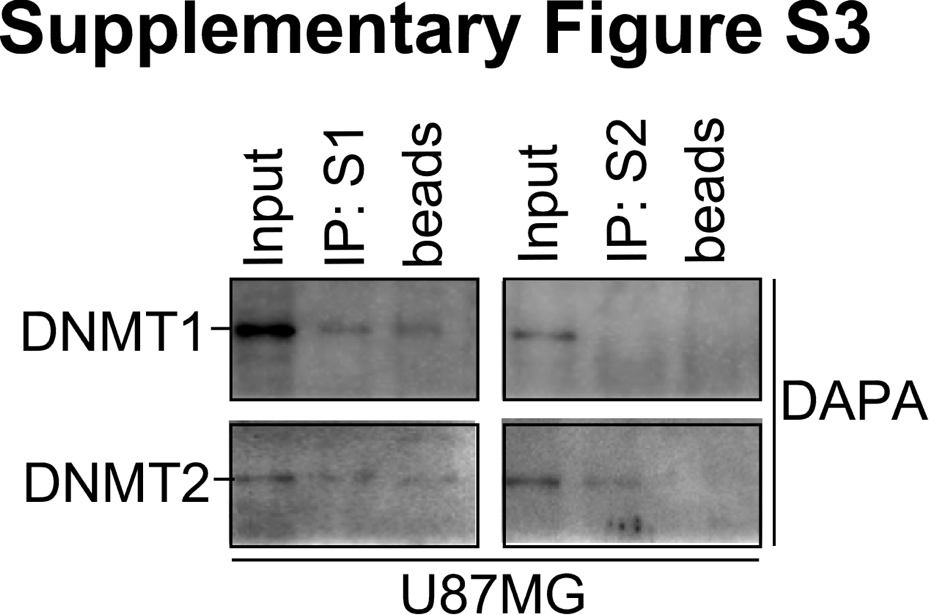


**Supplementary Figure S7. The association of CYP17A1 with DNMT1 and DNMT2 analyzed by DAPA assay.** After mixing with S1 or S2 probe, protein-DNA complex was precipitated by protein A agarose and analyzed by Western blotting.


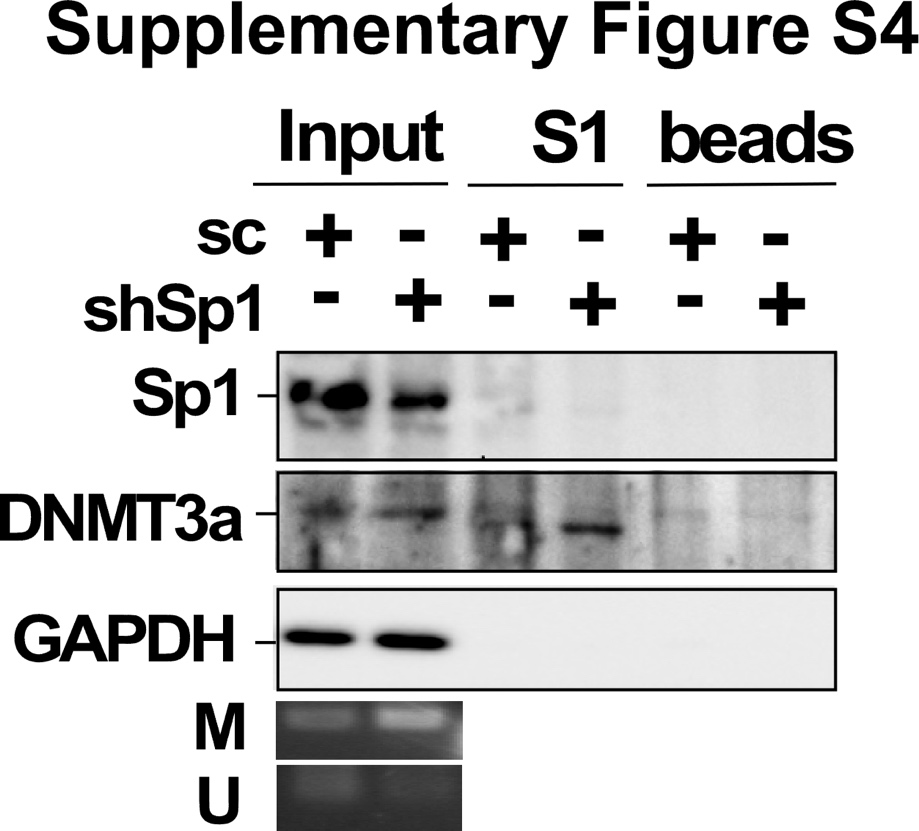


**Supplementary Figure S8. Sp1 knockdown increases the association of DNMT3a with the CYP17A1 promoter.** After Sp1 knockdown, cell lysates were prepared and mixed with the S1 probe. Agarose-precipitated protein-DNA complex was analyzed by Western blotting.


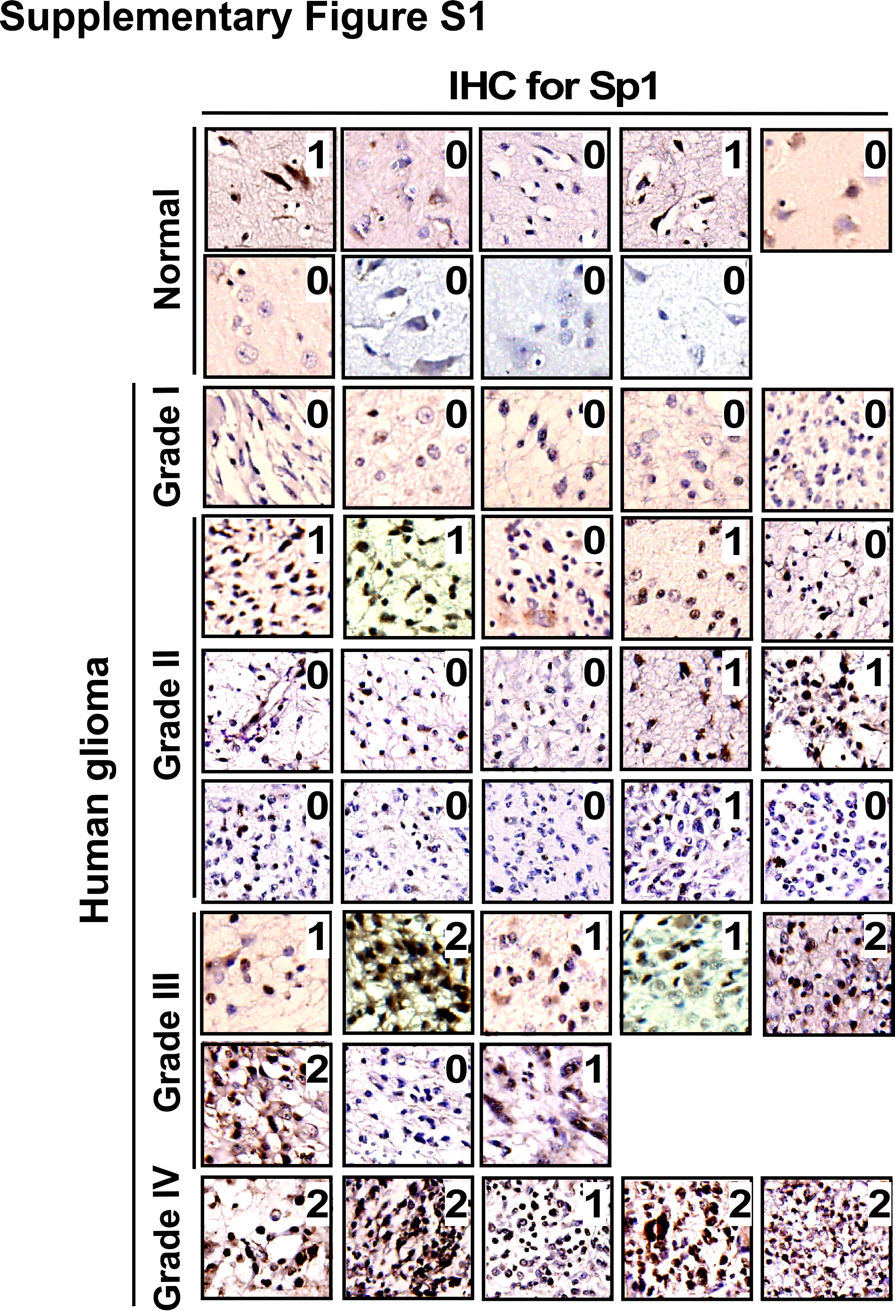


**Supplementary Figure S9. Sp1 expression in human glioma.** The glioma tissue array was immunostained by the anti-Sp1 antibody.


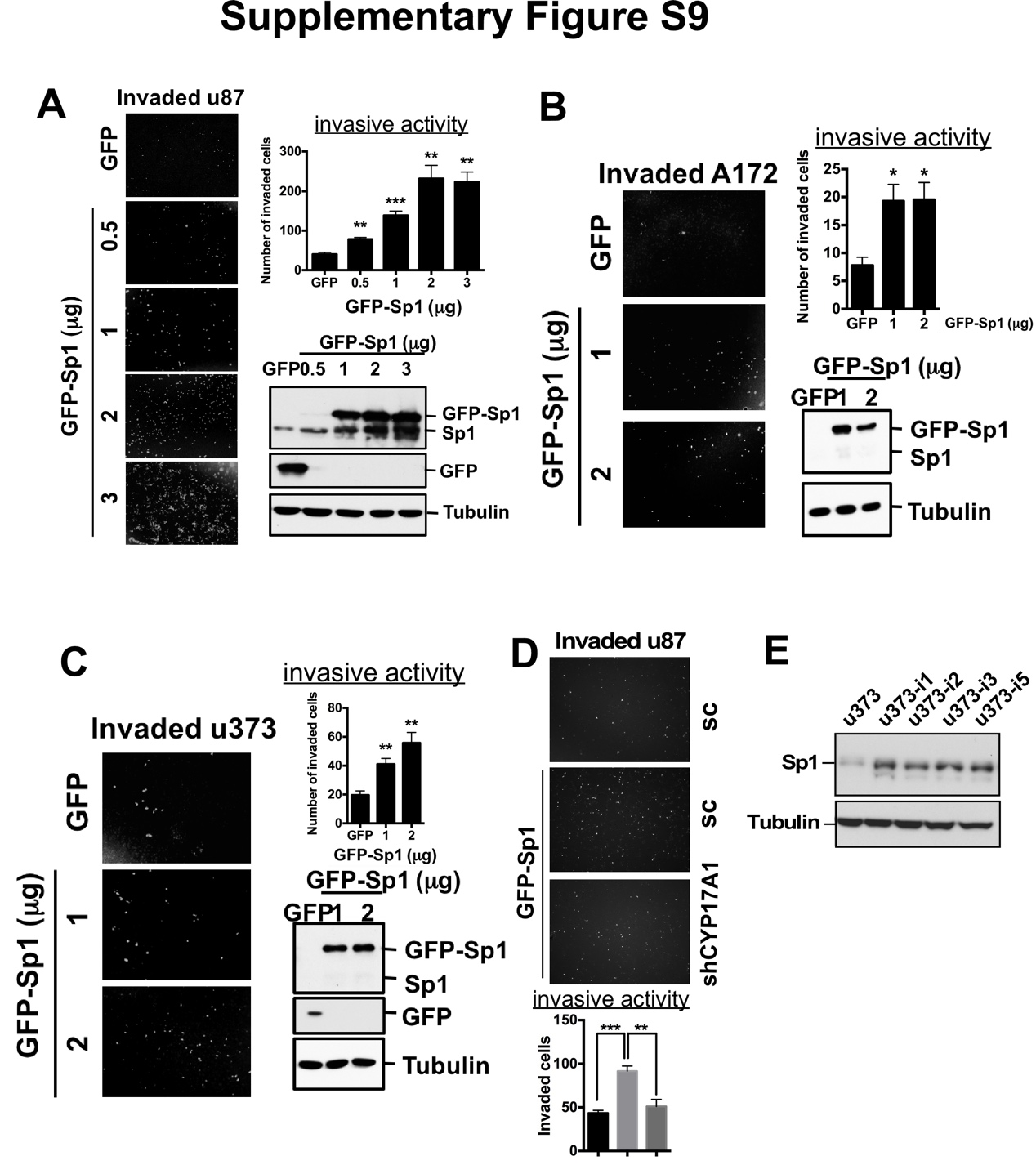


**Supplementary Figure S10. Sp1 promotes glioma invasion.** A, B, C. After transfection with GFP-Sp1, cells were subjected to matrigel-combined transwell invasion assay and Western blotting using indicated antibodies. D. After GFP-Sp1 overexpression and CYP17A1 knockdown, cells were harvested for invasion assay. E. Cell lysates were collected for Western blotting using the anti-Sp1 antibody.


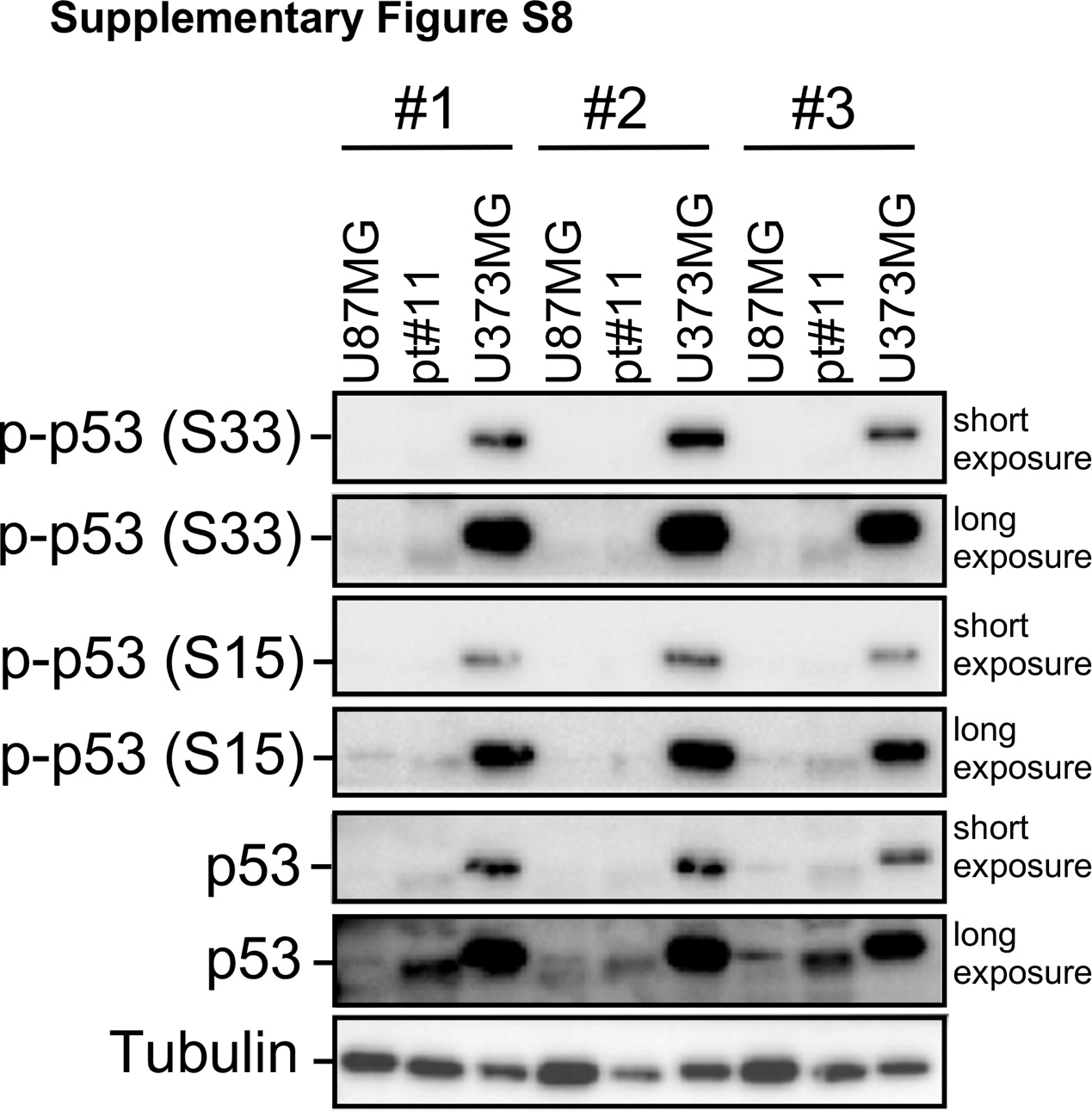


**Supplementary Figure S11. Expression and phosphorylation of p53 protein in glioma cells.** Total lysates of U87MG, pt#11 and U373MG were prepared for western blotting using the anti-p53, anti-p-p53-S15, anti-p-p53-S33 antibodies.

**Supplementary Table S1. Characteristics of glioma patients and non-cancer individuals**

|  | **Glioma** | **Non-cancer** |
| --- | --- | --- |
| **Gender (n)** |  |  |
| Male | 14 | 7 |
| Female | 12 | 8 |
| **Age (mean±s.e.m)** | 57.8±3.3 | 58.5±2.2 |
| **Grade (n)** |  |  |
| I | 6 |  |
| II | 6 |  |
| III | 4 |  |
| IV | 10 |  |
| **DHEA (nM)** | 23.1±0.64 | 20.89±0.51 |

**Supplementary Table S2. Sp1 expression is increased in grade IV GBM.**

**Reference**

1 Hsu TI, Lin SC, Lu PS, Chang WC, Hung CY, Yeh YM *et al* (2015). MMP7-mediated cleavage of nucleolin at Asp255 induces MMP9 expression to promote tumor malignancy. *Oncogene* **34:** 826-837.

2 Chuang JY, Wang YT, Yeh SH, Liu YW, Chang WC, Hung JJ (2008). Phosphorylation by c-Jun NH2-terminal kinase 1 regulates the stability of transcription factor Sp1 during mitosis. *Mol Biol Cell* **19:** 1139-1151.

3 Hung JJ, Wang YT, Chang WC (2006). Sp1 deacetylation induced by phorbol ester recruits p300 to activate 12(S)-lipoxygenase gene transcription. *Mol Cell Biol* **26:** 1770-1785.

-
